# Automation of cellular therapy product manufacturing: results of a split validation comparing CD34 selection of peripheral blood stem cell apheresis product with a semi-manual vs. an automatic procedure

**DOI:** 10.1186/s12967-016-0826-8

**Published:** 2016-03-16

**Authors:** Christiane Hümmer, Carolin Poppe, Milica Bunos, Belinda Stock, Eva Wingenfeld, Volker Huppert, Juliane Stuth, Kristina Reck, Mike Essl, Erhard Seifried, Halvard Bonig

**Affiliations:** Department of Cellular Therapeutics (GMP), German Red Cross Blood Service Baden-Württemberg-Hesse, Institute Frankfurt, Frankfurt, Germany; Institute for Transfusion Medicine and Immunohematology, Goethe University Medical Center, Frankfurt, Germany; Miltenyi Biotec GmbH, Bergisch-Gladbach, Germany; Department of Medicine, Division of Hematology, University of Washington, Seattle, WA USA

**Keywords:** Stem cell transplantation, CD34, Haplo-identical, Cell therapy, Allogeneic, Immunomagnetic, Automation, Good manufacturing practice, Clean room, CliniMACS, Prodigy

## Abstract

**Background:**

Automation of cell therapy manufacturing promises higher productivity of cell factories, more economical use of highly-trained (and costly) manufacturing staff, facilitation of processes requiring manufacturing steps at inconvenient hours, improved consistency of processing steps and other benefits. One of the most broadly disseminated engineered cell therapy products is immunomagnetically selected CD34+ hematopoietic “stem” cells (HSCs).

**Methods:**

As the clinical GMP-compliant automat CliniMACS Prodigy is being programmed to perform ever more complex sequential manufacturing steps, we developed a CD34+ selection module for comparison with the standard semi-automatic CD34 “normal scale” selection process on CliniMACS Plus, applicable for 600 × 10^6^ target cells out of 60 × 10^9^ total cells. Three split-validation processings with healthy donor G-CSF-mobilized apheresis products were performed; feasibility, time consumption and product quality were assessed.

**Results:**

All processes proceeded uneventfully. Prodigy runs took about 1 h longer than CliniMACS Plus runs, albeit with markedly less hands-on operator time and therefore also suitable for less experienced operators. Recovery of target cells was the same for both technologies. Although impurities, specifically T- and B-cells, were 5 ± 1.6-fold and 4 ± 0.4-fold higher in the Prodigy products (p = ns and p = 0.013 for T and B cell depletion, respectively), T cell contents per kg of a virtual recipient receiving 4 × 10^6^ CD34+ cells/kg was below 10 × 10^3^/kg even in the worst Prodigy product and thus more than fivefold below the specification of CD34+ selected mismatched-donor stem cell products. The products’ theoretical clinical usability is thus confirmed.

**Conclusions:**

This split validation exercise of a relatively short and simple process exemplifies the potential of automatic cell manufacturing. Automation will further gain in attractiveness when applied to more complex processes, requiring frequent interventions or handling at unfavourable working hours, such as re-targeting of T-cells.

## Background

The promise of automation of cell therapy processing is several-fold. A closed system might allow the process to be performed in a less strictly controlled environment than currently (class A in B or 100 in 1000), or for several processes to proceed concurrently within the same room of a cell factory. Automation is expected to be labor saving, and specifically for very complex processes it might add stability and convenience, for instance with respect to the timing of sequential process steps (especially at inconvenient hours). With respect to product/process quality, improved stability/predictability of outcomes was posited as a likely outcome; experience thus far does not unequivocally support this expectation [1–3]. Before automats will be programmed to perform very complex processes consisting of a sequence of orchestrated process steps, such as washes, stimulations, incubations, selections, etc., the individual building blocks must be programmed and put to the test. Moreover, for cost effectiveness of the automats, customers may expect to perform all standard processes on one device. Therefore, an immunomagnetic selection module was generated for the cell processing automat CliniMACS Prodigy (referred to as “Prodigy” throughout the remainder of the manuscript) to copy the CD34 “normal scale” selection module for the established semi-automatic CliniMACS Plus System, although performance of CliniMACS Plus is excellent, highly robust and not particularly challenging nor time-consuming for cellular therapy laboratories. The specific technical challenge of the overall relatively simple CD34 selection process is the near-homogeneous enrichment of stem cells with minimal contamination by non-target cells that can be achieved with magnet, column and reagent, components which are shared by both systems. The number of contaminating cells largely represents the device’s efficiency at flushing unwanted cells from the complex tubing systems and less so, non-specific immunomagnetic retention. Previous analyses comparing outcomes of CD34 selections with the “large-scale” process on Prodigy versus on CliniMACS Plus had suggested greater non-target cell reduction in CliniMACS Plus products [4, 5]. In light of these data, the manufacturer re-designed the process on Prodigy with the aim to further reduce non-target cells. At the same time, the process became scalable, as the “normal-scale” module was introduced which uses only one vial of CliniMACS CD34 reagent. This allows for magnetic labelling at reduced material costs for smaller cell products of not more than 600 × 10^6^ CD34+ cells and 60 × 10^9^ WBCs. The “normal scale” Prodigy process was tested here for the first time. By performing split validations, where cells from the same G-CSF mobilized apheresis product were concurrently manipulated with both devices, product variables affecting outcomes were controlled and direct comparison of process outcomes was for the first time facilitated. The quality of the processes is described by feasibility, process time and labor consumption, as well as CD34 recovery and the efficiency of immune cell depletion. The manuscript presents several novelties about the automatic Prodigy CD34 selection process, albeit each one modest, namely, split validation, first reported use of the re-designed “LP-34 Enrichment” process and first reported use of the “normal scale” module.

## Methods

### Donors and cells

Peripheral blood apheresis products from G-CSF mobilized healthy volunteer donors were obtained during aphereses where favourable donor-recipient weight ratio and mobilization efficiency enabled the extraction of a large excess of cells within the 300 min apheresis duration allowed per German guidelines. Two such aphereses were performed; the second one served as starting material for split runs two and three. Donor assessment was done as described [6]. Stem cell donation for these validations required donors’ written informed consent. The protocol was approved by the ethics committee of Goethe University Medical School (#468/13) and was performed in agreement with the Helsinki declaration in its current version. Aphereses were performed with Terumo BCT Spectra Optia devices as described [7, 8]. Collection targets were the same as for clinical routine, i.e. hematocrit <4 % and maximal WBC extraction at the possible expense of higher neutrophil and platelet contents in apheresis products [7, 8]. Three products containing 37, 46 or 54 × 10^9^ total WBC and 811, 916 or 1145 × 10^6^ total CD34+ cells were obtained. Products contained 8–16-fold more T-cells and 3–4-fold more B-cells than CD34+ target cells. Collections and selections were done in Q3 of 2015.

### Immunomagnetic selection

The CliniMACS Plus and Prodigy devices [3, 5, 9, 10], CliniMACS TS and Prodigy TS310 tubing sets, CliniMACS CD34 reagent (1 vial each) and CliniMACS PBS/EDTA buffer were obtained from Miltenyi Biotec (Bergisch-Gladbach, Germany). NaCl 0.9 %, H_2_O *ad. inj.* and human serum albumin (HSA) were from Baxter (Unterschleißheim, Germany). Both the CliniMACS Plus and Prodigy “normal scale” CD34 selection modules’ specifications are up to 60 × 10^9^ total leukocytes or 600 × 10^6^ CD34+ cells, and one vial of CD34 reagent (the same reagent for both devices) is used to enrich the CD34+ cells. CliniMACS Plus selections were performed according to local SOP which are identical with manufacturer-recommended protocols; the method was previously published [9]. Briefly, platelets were depleted by successive soft-spins, cells were incubated with CliniMACS CD34 Reagent (monoclonal CD34 antibody coupled to superparamagnetic nanobeads), washed to remove free antibody and then connected to a TS tubing system which was fitted on the CliniMACS Plus device. CliniMACS Plus subsequently automatically performed the column application, wash and elution steps. Prodigy selections used the same reagent; a TS310 tubing set was installed and all liquids were connected as prompted by the device. After starting the selection, the process was completely automatically guided by a release candidate version of the “LP-34 Enrichment” process for Prodigy software version 1.2.0, including both “large scale” and “normal scale” options. In view of the information gained during evaluation of the “large-scale” CD34 selection process, [4, 5] in Prodigy software version 1.2.0 “LP-34 Enrichment” “large scale” and “normal scale” processes were modified from the version used in the referenced work (1.1.4 or prior) to further reduce non-target cell trapping in the tubing system, by increasing the intensity of the washing steps of the separation column and pre-column. The “LP-34 Enrichment” process together with Prodigy software version 1.2.0 not yet being CE marked, none of the cell products were intended for clinical use.

### Assessment of selection outcomes

Leukocyte concentrations in starting population, non-target and target population were determined using the Sysmex XT1800 (Norderstedt, Germany) automatic hemocytometer. Flow cytometry was performed with FACSCalibur and LSRFortessa (Becton–Dickinson, Heidelberg, Germany). Cells were stained with the following antibodies (all from BD Biosciences unless otherwise noted): anti-CD45-FITC (2D1)/anti-CD34-PE (8G12) (BD Stem Cell Reagent), anti-CD14-V450 (MφP9), anti-CD3-APC (SK7), anti-CD4-AmCyan (SK3), anti-CD8-APC-Vio770 (BW135/80, Miltenyi Biotec), anti-CD20-APC-eFluor780 (2H7, eBioscience, Frankfurt, Germany), anti-CD56-PE-Cy7 (CMSSB, eBioscience). To assess viability, 7AAD (BD Biosciences) was added to the FACS suspension buffer. IVD grade reagents were used where possible. Three platforms each were tested on apheresis product and positive fraction, i.e. the commercial single-platform SCE-kit (BD), [11] our clinical routine single-platform residual T-cell detection panel, validated to detect 1 T-cell in 10 × 10^3^ non-T-cells with a precision of ±20 % (0.8–1.25 T-cells per 10 × 10^3^ non-T-cells), and a second residual cell identification panel designed for extended characterization of leukocyte subsets for the purpose of research/development studies such as this one, as previously reported [4]. Unless otherwise indicated, all cell concentrations, frequencies or numbers refer to 7AAD-negative (viable) cells only. In two split-validations, colony forming activity was assessed on the final products; aliquots of cells were plated in commercially available cytokine-replete semi-solid media (MethoCult H4434, Stem Cell Technologies, Vancouver, BC) and read after 2 weeks’ incubation under standard conditions, using an inverted microscope with 2.5× magnification, as described [11].

### Goals of the study

The aims of the study were to test feasibility of CD34 cell selection with the “normal scale” CD34 selection module on Prodigy and to compare type and quantity of contaminating non-target cells in Prodigy- and concurrently generated CliniMACS Plus-products. The pre-defined pass criteria for the validation exercise as outlined in the change control process was generation of three successive Prodigy products meeting the specification of an allogeneic CD34-selected product, for which our institution holds a marketing authorization. Besides being sterile and non-infectious with a panel of blood-transmissible agents, products must contain a dose of viable CD34+ cells of ≥4 × 10^6^/kg and ≤50 × 10^3^ T-cells/kg of the recipient (i.e. T-cell frequency cannot exceed 1.25 % of the CD34+ cell frequency). B-cell content must be measured and declared. This specification is based on a joint position paper by the German societies for Hematology/Oncology DGHO, Pediatric Hematology/Oncology GPOH and Transfusion Medicine DGTI and thus applies to all licensed allogeneic CD34 selected transplants in Germany.

### Statistics

Data were entered into Excel (Microsoft, Redmond, WA) spreadsheets from which descriptive statistics were extracted. −log T-cell depletion was calculated as the negative logarithm to base 10 of number of total T-cells in the final product divided by the number of T-cells in the apheresis product [12]. Student’s t test was used to identify statistically significant differences between CliniMACS Plus and Prodigy products; significance was assumed at p < 0.05.

## Results

### Process and process stability

Three concurrent CliniMACS Plus and Prodigy selection split-runs were performed; all were successful in that they proceeded without issues and generated highly pure CD34 products. At the start of the selection processes, apheresis products were 20–44 h old. For details on selections with CliniMACS Plus, please refer to the user handbook [13]. Briefly, platelets are removed by soft spins of the apheresis product in centrifuge bags, leukocytes are incubated with CD34 reagent, excess reagent is removed by successive washes, after which the cell suspension is applied to the CliniMACS Plus which performs an automatic column selection and elution into the target cell bag. Prodigy selections were facilitated by the user interface which guides the user through tubing set installation and cell and reagent connection. Priming and subsequently the selection process are initiated by pressing the start button, beyond which the process proceeds automatically. Other user interactions are no longer necessary. Including set-up, the Prodigy process takes in mean about 5 ½ h (the speed of instrument set-up significantly depends on the experience of the operator). In this split-validation exercise, the CliniMACS Plus runs were performed by three different operators who needed in mean 4 ½ h for the entire process including the platelet washes, thus besting Prodigy by about 1 h.

#### Product properties

**Fig. 1:**
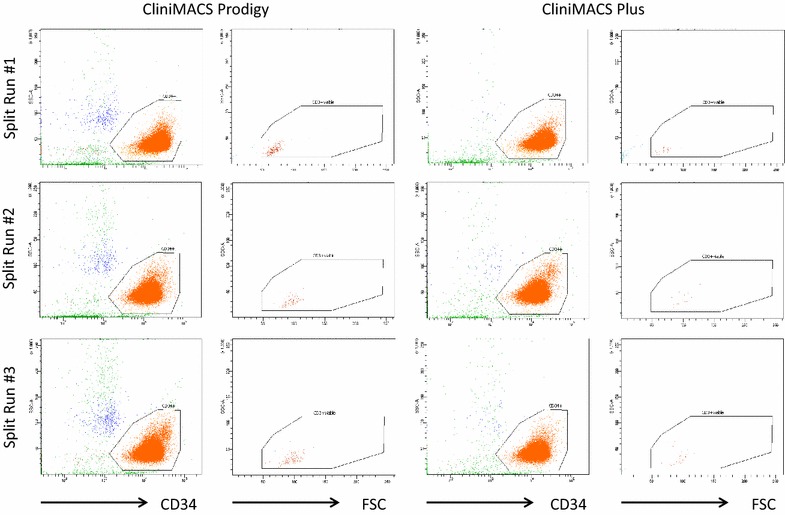
Flow cytometric appearance of CliniMACS Plus or Prodigy generated CD34 selection products: excerpts from the flow cytometric analysis (quality control) of the final products from all three runs are depicted; in all cases 100,000 CD45 + events (WBC) were acquired. For Prodigy and CliniMACS Plus, respectively, the *left panels* show SSC over CD34; besides CD34+ target cells (*orange*), SSC-intermediate, CD34-negative monocytes (*blue*) and within the lymphocyte region (SSC-low, CD34-negative), T-cells (*red*) are visible. The *right panels* depict CD45+ CD3+ events for Prodigy and CliniMACS Plus, respectively. The greater content of monocytes and T-cells in Prodigy products is readily visible. Corresponding numbers are given in Table 1

Three apheresis products containing in 133−200 ml 37−54 × 10^9^ leukocytes, 820–1145 × 10^6^ CD34+ cells and 7.3–13 × 10^9^ T-cells were evenly divided to serve as starting materials for simultaneous selection of CD34+ cells with the two systems. Properties of Prodigy and CliniMACS Plus CD34+ cell products (target population) are detailed in Table 1; briefly, recovery of target cells was similar with both (58 ± 5 vs. 60 ± 5 %, respectively) and purity exceeded 96 % CD34+ cells among total WBCs. In none of the six processes, the T-cell frequency relative to the CD34+ cells exceeded 0.25 %, thus easily meeting the pre-defined specification of ≤1.25 % in all cases. Outcomes differed, however, in the calculated depletion factor for T-cells (5.78 × 10^−5^± 8.63 × 10^−6^ vs. 1.32 × 10^−5^± 1.95 × 10^−6^, respectively, p = 0.033; 4.24 vs. 4.88 log) and for B-cells (3.52 × 10^−4^± 6.27 × 10^−5^ vs. 9.30 × 10^−5^± 1.51 × 10^−5^, respectively, p = 0.016; 3.45 vs. 4.03 log) in favour of CliniMACS Plus. Flow cytometric analyses of the target populations are shown in Fig. 1, illustrating discernibly greater numbers of non-target cells in the Prodigy products. Functionality of selected CD34+ cells was tested in colony assays from selections 2 and 3; similarly to unselected products, approximately 3.5 CD34+ cells gave rise to one CFU-C with no difference between selection methods.

**Table 1:** Product properties

	Split run #1		Split run #2		Split run #3	
	CliniMACS	Prodigy	CliniMACS	Prodigy	CliniMACS	Prodigy
Apheresis product						
Volume (ml)	70	66	100	100	80	80
WBC (×10^6^/ml)	269.8	272.6	268.3	268.3	287.4	287.4
WBC (×10^9^)	18.9	18	26.8	26.8	23	23
Hkt (%)	2.5	2.5	1.3	1.3	1.7	1.7
Plt (×10^6^/ml)	2740	2740	1934	1934	1971	1971
Plt total (×10^9^)	191.8	180.8	193.4	193.4	157.7	157.7
T-cells (×10^6^)	6818	6428	4587	4587	3670	3670
CD4+ (% of CD3 +)	70	70	58	58	58	58
CD8+ (% of CD3 +)	30	30	42	42	42	42
CD34+ cells (%)	2.2	2.2	2.1	2.1	2.1	2.1
CD34+ cells (×10^6^)	417.8	393.3	572.6	572.6	458.1	458.1
B-cells (×10^6^)	1813	1709	1550	1550	1240	1240
Non-target fraction						
Volume (ml)	298	393	328	389	301	394
WBC (×10^9^)	16.1	14.6	23.8	22.3	14.5	16.8
CD34+ cells (%)	0.2	0.2	0.2	0.2	0.3	0.2
CD34+ cells (×10^6^)	33	31	49	44	47	37
Target fraction						
Volume (ml)	40	78	41	82	39	76
WBC (×10^9^)	0.27	0.2	0.39	0.39	0.24	0.29
Plt (x10^6^/ml)	4	4	7	8	5	7
T-cells (×10^6^)	0.08	0.47	0.05	0.2	0.06	0.21
CD4+ (% of CD3 +)	50	50	9	43	11	24
CD8+ (% of CD3 +)	50	50	91	57	89	76
CD34+ cells (%)	97	96.1	99	98.2	98.8	98
CD34+ cells (×10^6^)	265.7	191	387.9	379.9	234	273.4
B-cells (×10^6^)	0.2	0.47	0.16	0.74	0.08	0.38
NK-cells (K/µl)	n.d.	n.d.	n.d.	n.d.	n.d.	n.d.
Monocytes (K/µl)	0.004	0.022	0.012	0.038	0.009	0.029
Ratio T:CD34+	3.01^−4^	2.45^−3^	1.27^−4^	5.18^−4^	2.67^−4^	7.73^−4^
T-cells/4 × 10^6^ CD34+	1204.5	9803.1	507.4	2072	1066.5	3090.6
B-cells/4 × 10^6^ CD34+	3011.1	9803.1	1691.4	7770.1	1333.1	5558.6
Recovery CD34+ (%)	63.6	48.6	67.7	66.3	51.1	59.7
B-cell depletion	1.10^−4^	2.74^−4^	1.06^−4^	4.76^−4^	6.29^−5^	3.07^−4^
T-cell depletion	1.17^−5^	7.28^−5^	1.07^−5^	4.29^−5^	1.70^−5^	5.76^−5^

## Discussion

The split-product validation exercise was performed under full GMP and using mobilized apheresis material of typical size for clinical products and results are thus directly transferrable to routine clinical processing. Clearly both methods are capable of generating the desired cell product, i.e. highly enriched CD34+ cells with fewer than 1.25 % T-cell admixture, which at least in Germany is the common specification of the allogeneic CD34+ stem cell (Table 2) product for use in mismatched (typically haplo-identical) transplantation. In two separate previous evaluations performance of Prodigy with prior Prodigy software versions (1.1.4 by Spohn et al. and, although not specified in the manuscript, an even earlier version by Stroncek et al.) was already compared to historical data, obtained with the CliniMACS Plus System [4, 5]. The data generated in this split validation were therefore used to support the application of the manufacturer of the system for approval of the CE mark from the national authority for the second, scalable version of the “LP-34 Enrichment” process offering both “normal scale” and “large scale” options CD34 process on Prodigy. The system consists of the reagents which are the same as are used for the CD34 process on the CliniMACS Plus, the Prodigy consumable (tubing set) and the new “LP-34 Enrichment process” on Prodigy software version 1.2.0.

**Table 2:** Comparative product quality (target fraction; mean ± SEM)

	CliniMACS	Prodigy	t test
T-cells/4 × 10^6^ CD34+	926 ± 213	4989 ± 2425	0.171
B-cells/4 × 10^6^ CD34+	2012 ± 1512	7711 ± 6664	0.013
Recovery CD34+	61 ± 5	58 ± 5	0.736
B-cell depletion	9.30^−5^ ± 1.51^−5^	3.52^−4^ ± 6.27^−5^	0.016
T-cell depletion	1.32^−5^ ± 1.95^−6^	5.78^−5^ ± 8.63^−6^	0.007

The fact that only “super-donors” were eligible for cell donations for this study implies comparatively high CD34+ cell frequencies and thus relatively favourable T-cell to CD34+ cell ratios in the apheresis products. Considering how –log T-cell depletion is calculated, the observed T-cell reduction with both systems is therefore quite satisfactory.

Work by Stroncek et al. [5] as well as our own previous work [4] with the “large-scale” CD34+ cell selection process had suggested slightly less efficient T-cell reduction on Prodigy than might have been expected based on clinical products processed with CliniMACS Plus, but as mentioned above, systematic differences between clinical and validation apheresis products did not allow for definitive conclusions. Our split validation now confirms statistically significantly less efficient immune cell depletion with the automatic device, although, as mentioned above, products meeting specification are generated by both devices. Center specific acceptance criteria for clinical stem cell products may be different from those used for this study and because of higher desired stem cell doses or lower tolerance for T-cells may require T-cell frequencies of less than 0.1 % among CD34+ cells (10 × 10^6^ CD34+/kg, 10 × 10^3^ T-cells/kg). 3 of 3 and 2 of 3 runs fulfil these more stringent requirements for CliniMACS Plus and Prodigy respectively, requiring a T cell depletion of more than 4.2 log with the CD34 and T cell frequencies of the leukapheresis products used in this study. Non-selected clinical stem cell products may differ considerably in composition, [9] possibly affecting separation performance additionally to impacts by design and tubing set of the system used for processing. In the largest published work to this topic, above-mentioned 4.2 logs T-cell depletion was achieved in 75 % of 139 CliniMACS Plus CD34 selected grafts [9]. With respect to generating CD34-selected stem cell products, larger studies will be required to evaluate whether the automat Prodigy results in a more consistent T cell depletion of more than 4.2 log than the currently used semi-automat CliniMACS Plus, although available data suggests that with the current software and consumable it might not. The lineage distribution of non-CD34+ cells in the final product (TARGET) differed somewhat from the starting population (ORI), in that T-cells were relatively more efficiently depleted (frequency among non-CD34+ MNCs threefold lower than in TARGET vs. in ORI) than B-cells (twofold higher) and monocytes (one-third higher), but with no striking differences between the selection methods. Thus Prodigy products contained approximately fivefold, fourfold and sevenfold greater numbers of residual T-cells, B-cells and monocytes than CliniMACS Plus products, i.e. non-target cell contamination was not specific or preferential for one lineage. While Prodigy does not reduce the total process time over CliniMACS Plus (on the contrary), the hands-on operator time is reduced by almost 2 h. Moreover, the Prodigy process could run unsupervised or supervised remotely over-night so that cells could be generated during unfavourable working hours. Thus selections could be routinely started on the apheresis day as opposed to the day thereafter, yielding a several hours fresher product. The earlier release of these products would then allow for transportation to transplant centers for same-day infusion. Avoidance of unfavourable working hours will become a more relevant issue with complex cell manipulation processes, as is already apparent with the automated process for enrichment of virus specific T cells [1] which no longer requires manual addition of peptide reagent between midnight and 2 am and will be even more accentuated as longer multi-step processes are developed on the automat. Automation of CD34+ cell selection also optimizes personnel and clean-room utilization, with the automat taking over the “second (unsupervised) shift” and by concurrently operating several automats within the same clean room. While few centers will generate sufficient CD34+ cell products to render this issue relevant for the process tested here, the same advantages will similarly apply to other, frequently requested cell products as may be established with the advent of T-cell products re-targeted against cancer antigens, as well as the availability of automatic systems may encourage centralization of cell selection processes.

Downsides, clearly, are the apparent superiority of the semi-manual process with respect to non-target cell contamination and the difficulties rescuing a partly processed product in case of a system failure.

## Conclusions

This is the first report on performing the “normal scale” CD34 selection with the “LP-34 Enrichment” process on the Prodigy. The Prodigy is unconditionally suitable to perform the CD34 selection process, in that all validation products met the pre-defined specification of the cell product “G-CSF mobilized allogeneic HPCs, CD34 selected”, a marketing authorization for which has been issued to several cell therapy laboratories in Germany, including the one the authors are affiliated with. Recovery of target cells was equal to that reported for the semi-automatic method. Final product content of potentially allo-reactive T-cells, as well as B-cells and monocytes was statistically significantly higher. Prodigy reduces operator time by approximately half and at least theoretically allows unsupervised, e.g. nocturnal, operation (although may be limited by liability issues). Although an automat like Prodigy is simple enough for any transplant unit to use (and a clean room facility may be dispensable for its operation), the quality control (residual T-cell quantification) is quite demanding and should be performed in centers of excellence. World-wide shipping from a very small number of centralized manufacturing sites is conceivable but may not be cost-effective.
